# Exploring public health equity in digital access: voices from marginalized groups using the “Mawid” application in Saudi Arabia

**DOI:** 10.3389/fdgth.2026.1917318

**Published:** 2026-09-16

**Authors:** Fahad M. Al-Anezi

**Affiliations:** College of Business Administration, Department Management Information Systems, Imam Abdulrahman Bin Faisal University, Dammam, Saudi Arabia

**Keywords:** access, application, care, digital health, digital-divide, health equity, healthcare, marginalized groups

## Abstract

**Background:**

Digitalization of healthcare services can significantly enhance the services and access to quality care. However, the equity implications of access to healthcare by such digital health platforms for marginalized populations remain underexplored, especially in the countries which are rapidly transforming their healthcare system through digitalization, such as Saudi Arabia. In this context, this study aims to investigate digital inclusion and health equity among users of the Saudi Ministry of Health's “Mawid” application.

**Methods:**

A qualitative exploratory design was adopted in this study using five focus groups discussions (25 participants with five participants in each group) as an approach for data collection. Each focus group is categorically included participants of older adults, rural residents, low-income individuals, people with disabilities, and intersectional users who combined these characteristics respectively. Braun and Clarke's thematic analysis framework was used to identify key patterns and themes from the data collected through discussions.

**Results:**

Findings revealed that Mawid often functioned as a conditional gatekeeper to healthcare, with barriers related to digital literacy, infrastructural constraints, time poverty, and accessibility. Participants relied heavily on informal support networks, frequently at the cost of privacy and autonomy. Uniform system design was perceived to disadvantage users with complex social and health needs.

**Conclusion:**

Government-managed digital health applications enhances efficiency of healthcare operations, but may reinforce health inequities, if equity-centred digital design and alternative access pathways are not considered in the system design and formulation of digital health strategies.

## Introduction

The rapid adoption of innovative technologies such as artificial intelligence (AI) within healthcare systems has significantly transformed the delivery and experience of healthcare, particularly in how individuals engage with health services, communicate with healthcare providers, access care, and self-manage their health conditions (1). While technological integration can improve the efficiency and effectiveness of healthcare services, it is equally important to assess whether individuals are prepared and able to adopt and use these systems. This raises important questions regarding equity and inclusion, which are fundamental principles of public health systems globally (2, 3). Digital health innovations should therefore not only improve healthcare efficiency and effectiveness but also ensure that individuals across different social groups, regardless of age, income, education, or geographic location, can benefit from them. Failure to ensure equitable access and participation may reinforce existing structural inequalities in health outcomes (4).

Digital inclusion refers to the ability of individuals and groups to access, understand, and effectively use digital technologies and is increasingly recognized as an important determinant of health equity (5). Conversely, digital exclusion reflects disparities in access to and effective use of digital technologies, including internet connectivity, devices, digital skills, and the ability to navigate complex e-health interfaces (6, 7). The multidimensional nature of the digital divide encompasses broader socioeconomic, geographical, cultural, and psychological inequalities and processes of marginalization (8, 9). Theories of technology-mediated inequality further suggest that when digital tools and applications are not inclusively designed and implemented, their benefits may disproportionately accrue to technologically proficient individuals while creating additional barriers for those with limited digital proficiency or other contextual constraints (10, 11). In healthcare, such disparities may reduce access to timely services and limit individuals’ autonomy in managing their health. Understanding these differential experiences is therefore essential for operationalizing health equity within increasingly digital and AI-enabled health systems.

**BOX 1:** Contributions to the literature.

• Shifts digital health assessment beyond usability and adoption toward equity, dignity, privacy, and lived social constraints shaping healthcare access. • Provides qualitative evidence from Saudi Arabia, addressing a regional gap in population health research on digital exclusion. • Informs public health policy by highlighting why uniform digital systems risk widening health inequities without parallel non-digital access pathways

Saudi Arabia provides an important context for examining these issues because of its rapid digital transformation of healthcare under Vision 2030 (12). Among the country's digital health initiatives is Mawid, a Ministry of Health application designed to facilitate appointment scheduling, referral management, telehealth consultations, and health education and awareness services (13). The application has achieved substantial population-level reach, with more than 24 million users, representing approximately 68% of the Saudi population (14). However, widespread adoption does not necessarily indicate equitable access or meaningful use across population groups. Older adults, individuals with lower incomes, and residents of rural or less-developed regions may encounter distinct structural, social, cultural, and psychological barriers to using digital health services. These barriers may include limited digital literacy, accessibility challenges, inadequate infrastructure, lack of technical support, and concerns regarding trust and usability. Thus, the high level of adoption of Mawid provides an important basis for examining whether the benefits of digital healthcare are distributed equitably across different population groups.

The concept of the digital divide has consequently evolved beyond simple differences in internet access to encompass digital literacy, usability, accessibility, trust, and the capacity to meaningfully engage with digital health services (15). Previous research has consistently identified older adults, low-income individuals, and rural residents as groups that may experience disproportionate barriers to digital health adoption (16, 17). Among older adults, reduced digital confidence, increased cognitive load, and accessibility limitations may hinder the use of mobile health applications (18). Low-income populations may experience constraints related to reliable internet access, device ownership, and digital health literacy (19), while rural populations may face limitations in digital infrastructure and access to technical support (20). Although digital health platforms can improve healthcare utilization, continuity of care, and patient autonomy among digitally capable users (21), they may simultaneously create barriers for those who are unable to use them effectively, particularly when digital pathways replace rather than complement traditional routes to healthcare access (22). This concern is especially relevant for government-provided digital health services that, although formally voluntary, may become strongly encouraged or functionally necessary for accessing healthcare.

Existing evidence concerning Mawid has largely focused on awareness, usability, satisfaction, and perceived effectiveness. Several studies reported high levels of user satisfaction and usability, particularly during the COVID-19 pandemic (13, 23, 24). However, these findings provide limited insight into whether the application's benefits have been distributed equitably, particularly in the post-pandemic period. Higher awareness of Mawid functionalities has been associated with greater likelihood of use (25), while differences in awareness have been reported according to age, income, employment status, education, and geographic region (25, 26). Older adults, lower-income individuals, and residents of less-developed regions have been reported to demonstrate lower levels of knowledge and independent use and to rely more frequently on family members to access services through the application (26). Although studies have also identified low digital literacy, unreliable internet access, and limited trust as barriers to telehealth adoption among older Saudis (27, 28), geographic isolation and limited primary healthcare infrastructure remain important challenges in rural areas (29, 30). Importantly, much of the existing research has relied on technology acceptance or user-experience frameworks, with comparatively limited application of digital inclusion and health equity perspectives. Consequently, structural barriers related to digital literacy, accessibility, usability, trust, and infrastructure remain insufficiently examined in relation to equitable use of Mawid (25).

Therefore, despite the extensive adoption of Mawid and the growing body of research examining its usability and acceptance, an important knowledge gap remains concerning whether the application supports equitable access and meaningful participation across socially and geographically diverse population groups. In particular, limited evidence is available regarding how marginalized users experience and navigate the platform and which structural and individual-level factors facilitate or constrain their effective use. Addressing this gap is important for ensuring that the ongoing digital transformation of the Saudi healthcare system contributes to, rather than exacerbates, existing health inequalities. Accordingly, this study aims: (1) to examine the extent of digital inclusion and equitable access to the Mawid application among marginalized user groups in Saudi Arabia, including older adults, low-income individuals, and rural residents; and (2) to identify and analyze the key barriers and facilitators affecting these groups’ ability to effectively use the Mawid system, with particular focus on digital literacy, usability, accessibility, and perceived trust in the digital health platform.

## Methods

### Study settings and participants

A descriptive qualitative design was adopted in this study to gain a deeper understanding on digital access and health equity in the use of the Saudi Ministry of Health's Mawid application among underrepresented populations. A qualitative approach is adopted because assessing multi-dimensional constructs such as health equity and digital divide, which is associated with social, cultural, psychological, and technical aspects is complex and context-dependent, which may not be effectively analyzed using quantitative approaches, as they require deeper interpretation of users’ perceptions (31, 32). Therefore, focus group discussions, discussions were adopted in this study, which facilitates in-depth exploration of participants’ lived experiences, perceived barriers, and enabling factors influencing equitable engagement with the Mawid platform. Accordingly, participants were included if they were 50 years of age or older for the older-adult group, or if they belonged to one of the targeted marginalized categories: rural residents, low-income individuals, people with disabilities, or intersectional users. For the purpose of this study, “older adults” were defined as individuals aged 50 years and above. Although international and Saudi policy definitions often use thresholds of 60 or 65 years, several digital-health and technology-adoption studies in Saudi Arabia and similar contexts have used 50 + as a pragmatic cutoff to capture those beginning to experience age-related barriers in accessing eHealth services (33). In developing regions, the WHO has noted that “older adult” may correspond to ages 50–55 and above, reflecting earlier onset of functional aging, chronic conditions, and retirement transitions compared with high-income settings. In the Saudi and broader Gulf context, individuals in their early fifties frequently experience transitions associated with aging, including early retirement, onset of multiple chronic conditions, and reduced digital confidence, which may affect their engagement with digital health platforms (34, 35). In all cases, participants had to have used or attempted to use the Mawid application and possess at least basic familiarity with it. Individuals were excluded if they had never used or attempted to use Mawid, were unable to provide informed consent, or could not meaningfully participate in the focus group discussion because of severe communication, cognitive, or health-related limitations.

### Recruitment and sampling

A purposive sampling strategy (36) was adopted to ensure the inclusion of diverse participants from marginalized groups who could provide rich, context-specific insights into digital access and health equity in relation to the Mawid application. Potential participants were identified and invited through multiple channels to reach older adults, rural residents, low-income individuals, people with disabilities, and users with intersecting vulnerabilities. These channels included primary healthcare centers and clinics in urban, semi-urban, and rural areas; community organizations and social-support groups working with older adults and people with disabilities; and personal and professional networks of the research team. In addition, snowball sampling was used, whereby initial participants were asked to refer other eligible individuals they knew who had used or attempted to use the Mawid application.

Individuals were initially approached by the researcher or a research assistant, who provided a brief verbal explanation of the study and screened for eligibility based on the inclusion criteria: age ≥50 years for the older-adult group; residence in rural or remote areas; self-reported low income; presence of a disability; or a combination of these characteristics, together with having used or attempted to use the Mawid application. Those who expressed interest and met the eligibility criteria were provided with further information about the study and invited to participate in an online focus-group discussion. Participants were not recruited directly through the Mawid application or Ministry of Health databases. Recruitment was conducted independently of the Mawid system, although all participants were required to have some prior experience (actual use or attempted use) of the application. This approach was chosen to avoid restricting participation only to current registered users who actively engage with the system, and to include individuals who had tried but discontinued use.

Variation was sought in gender, age group, educational level, and region to capture diverse perspectives. An estimated sample size of 19 was identified. A final sample of 25 was achieved, who were grouped into five focus groups with five participants in each group. Focus groups were designed to be relatively homogeneous in terms of key demographic characteristics (e.g., age, residence, socioeconomic status, or disability status) to promote open discussion, while heterogeneity was maintained across groups to enable comparative analysis. Data collection continued until data saturation was reached, which occurred after the fifth focus group, as no new themes or substantial new insights emerged in the final discussions.

The recruitment strategy may have introduced some sample bias. Participants were recruited through clinics, community organizations, personal and professional networks, and snowball sampling, which may have favored individuals who already have some level of social connectedness, access to health services, or contact with community structures. Individuals who are completely disconnected from health facilities, community organizations, or social networks may therefore be underrepresented. In addition, because all participants were required to have at least some experience with the Mawid application, the sample does not include people who have never heard of or attempted to use the system. These limitations should be considered when interpreting the findings, particularly regarding the most digitally and socially excluded populations.

### Data collection

Focus group discussions were conducted through secure online video conferencing application from 8/12/2025 to 12/1/2026. Focus group discussions were conducted through a secure online video-conferencing application. This mode of data collection was selected due to the wide geographic dispersion of the target participants across urban, semi-urban, rural, and remote areas, which made in-person meetings logistically challenging and costly. Online discussions also offered greater flexibility in scheduling, reduced travel burdens for participants, and aligned with infection-control considerations in the post-pandemic period. To facilitate participation, individuals received simple written and verbal instructions on how to join the session, and were offered a brief technical check prior to the discussion. Where necessary, participants were allowed to join using a mobile phone with audio-only capability, and some participants were supported by a family member to connect to the session. Average discussion time ranged between 60 to 80 minutes. Discussions were guided by questions focusing on key components, which include the experiences of accessing and using Mawid, perceived usability and satisfaction, barriers to digital access and equity, trust and inclusion, and recommendations for improvement. The discussions were audio-recorded and conducted in Arabic with the consent of participants.

### Data analysis

All focus-group discussions were audio-recorded in Arabic and transcribed verbatim in Arabic by the author. The Arabic transcripts were then translated into English by the author, who is fluent in both Arabic and English. To ensure accuracy and preserve the meaning of participants’ expressions, a two-stage verification process was applied.

First, a bilingual expert independently reviewed all transcripts and compared the English translation against the original Arabic for accuracy, completeness, and nuance. Discrepancies and alternative interpretations were discussed and resolved by consensus, and the agreed approach was then applied consistently to the final English versions.

Second, for key illustrative quotes used in the Results, a back-translation procedure was employed: selected quotes were translated from English back into Arabic by a different bilingual researcher who was not involved in the initial translation. These back-translations were compared with the original Arabic transcripts to check whether the core meaning and emotional tone were preserved. Minor adjustments were made to the English wording where necessary to improve fidelity to the original expressions while maintaining readability. All final quotes used in the manuscript were checked against the original Arabic transcripts to ensure that they accurately reflected participants’ intended meanings.

Thematic analysis, following Braun and Clarke's six-phase framework (37), which includes familiarization, initial coding, theme development, review, definition, and reporting, was used to analyze the focus-group discussion data. An inductive coding approach was used for identifying the themes to naturally emerge from the focus-group discussion data by organizing patterns into codes and sub-codes. NVIVO, V.14 was used for data organization, coding, and theme refinement.

### Ethical considerations

Ethical approval was obtained from the Institutional Review Board of Imam Abdulrahman Bin Faisal University, Saudi Arabia (approval number: IRB-2025-14-0850). The study involved five online focus-group discussions with 25 participants. Before each discussion, participants received information about the study purpose, procedures, potential risks, confidentiality protections, the voluntary nature of participation, and their right to withdraw at any time without consequences. Verbal informed consent was obtained from all participants before data collection and was audio-recorded as part of the study documentation. All procedures were conducted in accordance with the ethical principles of the Declaration of Helsinki.

## Results

Participants reflected a diverse range (see Table A1 in Supplementary File), with each focus group including similar participants; and heterogeneity is maintained across the focus groups. Each focus group is categorically included participants of older adults, rural residents, low-income individuals, people with disabilities, and intersectional users who combined these characteristics respectively. Participants age group ranged from 29 to 71 years; representing both genders from urban, semi-urban, rural, and remote rural settings. Occupations varied across participants, such as retired workers, informal laborers, caregivers, and single parents. Low or uneven digital literacy, unstable work patterns, shared or basic devices, and health or functional limitations was observed among the participants reflecting the marginalized group. Thematic analysis of focus group discussions led to the identification of seven key themes (see Table A2 in Supplementary File), which are presented below.

### Apps as barriers to getting healthcare

The Mawid application was perceived across all focus groups as a barrier or gate that must be successfully crossed in order to receive care, framing access to healthcare as a conditional process dependent on digital competence. Older adults described anxiety and stress when using the application, fearing that a single mistake could lead to lost appointments and longer waiting times.

“One wrong press and the appointment is gone; then you wait again for weeks,” said an older participant (OA2).

“I feel scared to open the app; I always think I will make a mistake and lose my turn,” explained another older woman (OA4).

Low-income participants highlighted that the application disproportionately benefits those with good devices and stable connectivity, while impeding those with limited resources.

“If your phone is old or the net is slow, the app just keeps loading and you miss the appointment time,” said a low-income participant (LI3).

These issues resulted in delayed or irregular care, as users lacked confidence and feared being blamed for errors. Many participants contrasted the app with face-to-face or telephone booking, where they could explain their situation and sense of urgency to a receptionist.

“On the phone you can tell them it is urgent. The app does not listen,” noted a caregiver (LI5).

Participants challenged the notion that digital systems ensure equal treatment, arguing that “equality is not sameness” when starting points are unequal.

### Mismatch between the application design and lived realities

Participants highlighted a clear incompatibility between the application's design logic and their everyday realities in terms of time, work, and health. Low-income participants described frequent trade-offs between clinical attendance and income, as daytime and working-day appointments conflicted with their job schedules.

“The appointment times are for people who sit in offices, not for those who work all day in the heat,” remarked a delivery driver (LI2).

Single parents reported that rigid appointment rules, combined with childcare and school demands, made missed appointments feel like a moral failure rather than a consequence of structural constraints. Older adults with chronic illnesses such as diabetes and hypertension described the multi-step process and small fonts as overwhelming.

“When you are sick and tired, you cannot follow all these steps. The letters are too small,” said an older participant with chronic illness (OA4).

Rural participants noted that farming seasons and long travel times to clinics contradicted with strict booking windows, and that missing an appointment due to network failure or harvest work could have serious health consequences.

“When I am in the field during harvest, I cannot keep checking the phone for messages,” said a rural farmer (RR1).

### Layered infrastructural and contextual barriers

Infrastructural barriers constituted a second layer of exclusion, exacerbating existing geographic and economic disadvantage. Rural participants emphasized that the application is fully dependent on network quality, describing instances of the app hanging, missed notifications, and ambiguous appointment confirmations.

“Sometimes I travel all the way to the clinic, and they say there is no appointment in the system. The app said it was confirmed,” reported a rural participant (RR5).

These issues increased uncertainty and costs, with wasted trips resulting in loss of time, money, and work. Low-income participants noted the need to purchase more advanced mobiles to cope with these issues, adding substantial costs.

“We had to buy a new phone just for the app; for us this is a big cost,” said a low-income caregiver (LI5).

Intersectional participants described the transformation of healthcare access problems from “distance only” to “distance and technology,” reflecting an increased burden (MX1, MX4).

### Reliance on informal support and loss of privacy

Across all focus groups, most participants relied heavily on informal support networks to access the application, including family members, neighbors, friends, co-workers, and occasionally clinical staff. Older adults and people with disabilities often depended on children or siblings to schedule appointments, interpret notifications, and correct errors.

“I have to ask my son to book for me, even for private problems. It is embarrassing,” shared an older man (OA1).

This dependency was experienced as embarrassing and dignity-eroding, as sensitive health information became visible to others. Rural participants described sharing phones and asking for help as a necessity that compromised their privacy.

“I don't like that my Neighbour sees my messages when she helps me. It is not private,” said a rural homemaker (RR2).

Participants distinguished between “informal” support, which was *ad hoc* and contingent on goodwill, and “structural” support, which they felt should be built into the health system. This dependence on informal networks was linked to fragile trust; participants trusted the government but distrusted the application, which they felt undermined their realities.

### Accessibility, disability, and invisible exclusion

Participants with disabilities viewed accessibility as a major barrier, citing problems with design features. Users with vision problems reported difficulties with small text, poor contrast, and incomplete screen-reader compatibility.

“The letters are too small; even with my glasses I cannot read them,” said a participant with low vision (PD1).

Users with hearing difficulties noted that help materials lacked captions, while those with cognitive or learning challenges highlighted the cognitive load imposed by the multi-step process (PD4, PD5).

“The instructions use difficult medical words; I feel stupid when I don't understand,” reported a participant with low literacy (MX4).

These findings reflected that neutral and standard design features may not fit all users. As a result, disabled participants often relied on family or untrained clinic staff to access services. Participants identified the need for accessibility features such as screen-reader compatibility, adjustable text, simplified language, captions, and assisted booking options, viewing these changes as essential to equity rather than optional enhancements.

### Fragile trust and perceived misalignment of equity

Participants expressed confidence in the government's intentions but raised concerns about the application's ability to provide equitable care. A perceived divide was observed between urban residents, who benefited from stable infrastructure and swift bookings, and rural users facing network failures and travel burdens.

“They think everyone has good internet and a new phone. That is not our life,” said a remote rural participant (RR5).

This misalignment contributed to trust erosion and shaped perceptions of equity. Many participants felt that the application promoted access for the digitally privileged while creating barriers for marginalized users.

“It promotes efficiency, not equity. Those are not the same,” noted a rural participant (RR4).

This theme highlights that trust in digital health applications depends not only on institutional credibility but also on perceived fairness and responsiveness to lived diversity.

### Recommendations for inclusive design

Participants offered practical suggestions reflecting the concept of equity as designing around real lives rather than idealized, stable users. Recommendations included simplified or separate versions of the application for older adults, offline or SMS-based appointment scheduling, grace periods for missed appointments due to connectivity failures, and the preservation of in-person or telephone booking as parallel routes.

“There should be a simple version for old people, with big buttons and clear words,” suggested an older participant (OA3).

“If the net fails, there should be a way to confirm by SMS or phone, not only the app,” proposed a rural resident (RR2).

Some participants recommended community-based digital support points at clinics or neighborhood centers, providing in-person support and training. Findings suggested that true inclusion and health equity require design considerations for diverse user capabilities and circumstances, and warned that eHealth applications risk becoming mechanisms of digital exclusion if they prioritize efficiency over accommodating varied capacities and challenges.

Although most participants described Mawid as a barrier or conditional gatekeeper, a few reported more positive experiences. One older participant with moderate digital skills (OA1) stated that the app “*saves time and I do not need to talk to the receptionist*,” and preferred digital booking over face-to-face contact. Similarly, one rural school administrator (RR3) with moderate digital skills and a stable connection described the system as “*mostly easy*” and reported few missed appointments. These deviant cases highlight that digital exclusion is not uniform; individuals with better devices, connectivity, and digital confidence may experience Mawid as convenient rather than exclusionary. Nonetheless, these participants were in the minority, and the dominant pattern across groups was one of conditional access and added burden.

## Discussion

This study provides an understanding on how government facilitated digital health applications operate in practice among the marginalized population in the context of health-equity-centered focus; thereby extending the previous research in such applications like Mawid, which has largely focused on usability, satisfaction, and adoption metrics. Although, previous studies (13, 23–28) reported high acceptance and perceived usefulness of Mawid, mostly during the Covid-19 pandemic period, findings in this study revealed that digital access to healthcare applications function as a conditional gatekeeper for receiving care, disproportionately disadvantaging older adults, rural residents, low-income individuals, and people with disabilities. Therefore, it is evident that digital transformation can unintentionally lead to structural inequalities in certain contexts as marginalized populations, when access routes (requirement of digital skills and awareness) become mandatory rather than complementary (4, 8, 22).

Participants, especially older adults highlighted experiences of fear, anxiety, delayed care, and avoidance behaviors with the Mawid application, suggesting that lack of digital skills and cognitive load can lead to reduced digital confidence, which in turn can affect users engagement and access to care (18, 29). This finding aligns with multidimensional digital divide theories, where exclusion stems not just from access but from usability, literacy, and contextual fit (38–41). Furthermore, the findings in this study reflected that such barriers are not just individual skill deficits, but are reinforced by rigid application design which penalize errors such as delayed care, and removes opportunities for negotiation or explanation, which are essential for marginalized groups, such as older adults. In addition, socio-economic disadvantage was linked to limited digital engagement, particularly among the low-income participants as observed in (19), and further extended this by revealing the hidden costs of digital health applications, especially time poverty, data affordability, and job insecurity, which are rarely captured in previous empirical studies.

Focusing on the rural participants experiences, findings in this study reinforce previous observations in relation to infrastructural inequities and geographic isolation (20, 29, 30), but conceptualizes such barriers as layered exclusions, where digital applications add additional burden on top of existing barriers such as distance-related constraints in healthcare access. One of the key observations was access to healthcare was facilitated by informal support networks such as friends, family, colleagues across all the groups, highlighting the family dependence (26) in Saudi Arabia. However, these findings were extended by revealing that this dependency was viewed as dignity-eroding and privacy-compromising, highlighting an overlooked equity trade-off in digital health adoption.

Furthermore, the findings in this study strongly align with technology-mediated inequality theories (10–12), by reflecting how neutral or common system designs privilege users with digital skills and stable resources; while undermining the marginalized populations’ struggles. It was also observed that trust in Mawid application was largely institutional rather than technological, reflecting that trust is relational and contingent on perceived fairness and responsiveness (7, 28). Overall, the findings suggest that efficiency-driven digitalization risks undermining public health equity unless inclusive design and alternative access pathways are systematically embedded.

### Theoretical and practical implications

Theoretically, this study demonstrates the drawbacks in equality-based digital designs in healthcare by demonstrating that such designs can result in inequitable outcomes, reinforcing technology-mediated inequality theories. The findings reiterate that deficits in digital literacy is a failure in system design, rather than users drawbacks or shortcomings. Furthermore, it reiterates the multidimensional nature of digital divide and health equity factors, where emphasis should be given to both application's efficiency and also the users’ expectations, and accessibility issues. Focusing on the practical implications, the findings reflect the need for operationalizing equity-oriented digital health governance, including parallel non-digital access routes, simplified interfaces for older adults, accessibility-by-default design, and flexible appointment policies. System designers and policy makers should consider the lived experience feedback and the reality on ground, for designing digital health strategies for digital transformation in healthcare, to promote health equity by enhancing access to care, without pre-conditions and undermining marginalized vulnerable populations.

### Study limitations and future research

This study has several limitations. Firstly, the qualitative design and purposive sampling techniques may limit the generalizability of the findings beyond similar marginalized populations in Saudi Arabia. Secondly, data collection using online focus groups may have excluded individuals with the most severe digital access barriers. Although the study specifically targeted marginalized groups, all participants needed a minimum level of digital access (an internet-connected device and sufficient connectivity) and basic digital literacy, or the support of someone who could help them connect, in order to participate. This means that individuals without any reliable internet access, without a suitable device, or with extremely low digital confidence were likely underrepresented or entirely absent from the sample. The findings therefore reflect the experiences of marginalized users who are able to engage at least minimally with digital platforms, rather than those who are completely digitally excluded. Thirdly, data collection using online focus groups may have excluded individuals with the most severe digital access barriers, including those without reliable internet connectivity, appropriate devices, or sufficient digital literacy to participate in video-conferencing. Although the study specifically focused on marginalized groups, participants still needed a minimum level of digital access and support to join the discussions, which may have led to the exclusion of the most digitally excluded individuals. Fourthly, findings are based on self-reported experiences and perceptions, which might be influenced by social desirability and psychological factors. However, the study provides rich, contextual insights that are essential for understanding equity implications, which were often undermined in previous empirical studies. Future studies may adopt quantitative methods focusing specifically on marginalized populations with larger samples to further analyze the health equity dimension in the context of rapid digital transformation in the Saudi Arabian healthcare sector. Mixed-methods designs that combine surveys with in-depth interviews or focus groups could also help capture both the breadth and depth of digital exclusion and its impact on access to care.

## Conclusion

This study has found that the government-managed healthcare applications like Mawid have improved healthcare efficiency, but their equity implications remained uneven. Digital healthcare access for marginalized users is shaped by the intersection of social, economic, geographic, and functional constraints, which has transformed technology into a barrier rather than facilitator of quality and continued care. Therefore, in order to achieve health equity, there is a need to shift from traditional uniform digital health solutions and strategies, to an inclusive, flexible, and human-centered application design. By undermining such adjustments, national digital health applications may reinforce existing inequalities, rather than enabling sustained and enhanced access to quality care, as a part of healthcare digitalization.

## Data Availability

The raw data supporting the conclusions of this article will be made available by the authors, without undue reservation.

## Supplementary material

**Supplementary Table A1**

Participants demographics

**Supplementary Table A2**

Emergence of themes
