# Supplementary material for: Exploring public health equity in digital access: voices from marginalized groups using the “Mawid” application in Saudi Arabia
Source: Front Digit Health. 2026 Sep 16;8:1917318. doi: 10.3389/fdgth.2026.1917318 (PMC13625048; doi:10.3389/fdgth.2026.1917318)
Supplement: Supplementary file 1 [file Supplementaryfile1.docx]

# Appendix A

Table A1. Participants demographics

| **Participant ID** | **Focus Group** | **Gender** | **Age** | **Residence** | **Socioeconomic / Occupational Status** |
| --- | --- | --- | --- | --- | --- |
| OA1 | Older adults | Male | 62 | Urban | Retired teacher |
| OA2 |  | Female | 58 | Urban | Homemaker |
| OA3 |  | Male | 71 | Semi-urban | Retired government employee |
| OA4 |  | Female | 65 | Urban | Not employed |
| OA5 |  | Male | 54 | Urban | Taxi driver |
| RR1 | Rural residents | Male | 47 | Rural | Farmer |
| RR2 |  | Female | 52 | Rural | Homemaker |
| RR3 |  | Male | 38 | Rural | School administrator |
| RR4 |  | Female | 45 | Rural | Caregiver |
| RR5 |  | Male | 60 | Remote rural | Retired laborer |
| LI1 | Low-income | Female | 34 | Urban | Domestic worker |
| LI2 |  | Male | 41 | Urban | Delivery driver |
| LI3 |  | Female | 29 | Urban | Unemployed |
| LI4 |  | Male | 50 | Urban | Security guard |
| LI5 |  | Female | 45 | Urban | Part-time cleaner |
| PD1 | Disability | Male | 40 | Urban | Office clerk |
| PD2 |  | Female | 36 | Urban | Not employed |
| PD3 |  | Male | 55 | Semi-urban | Not employed |
| PD4 |  | Female | 48 | Urban | Not employed |
| PD5 |  | Male | 30 | Urban | Not employed |
| MX1 | Intersectional | Female | 55 | Rural | Informal worker |
| MX2 |  | Male | 63 | Urban | Retired |
| MX3 |  | Female | 42 | Urban | Not employed |
| MX4 |  | Male | 35 | Rural migrant | Daily wage worker |
| MX5 |  | Female | 50 | Urban | Intermittent employment |

Table A2. Emergence of themes

| **Major theme** | **Subthemes** | **Codes** | **Example statement** | **Example groups where prominent** |
| --- | --- | --- | --- | --- |
| 1. Apps as barriers to getting healthcare | Fear and anxiety in use; delayed/forgone care; equality vs equity mismatch | Fear of "making mistakes"; bookings as "tests"; "locked out" of care; "treats everyone the same" | "If you make one mistake, the system doesn't forgive you. That makes me nervous every time I open it." (OA1) | Older adults, low-income, intersectional |
| 2. Mismatch between app design and lived realities | Rigid rules vs real-life constraints; illness/fatigue impacts; temporal conflicts | Misalignment with shift work/caregiving/farming; no flexibility for health fluctuations | "When my blood sugar is low or when I'm tired, I struggle to concentrate. The letters are small, and there are too many steps." (OA4) | Older adults, low-income, rural, intersectional |
| 3. Layered infrastructural and contextual barriers | Network/device instability; hidden costs of digital ecosystem; compounded geographic barriers | App freezing/missing confirmations; data/charging costs; "distance + technology" problem | "Sometimes I try to book an appointment, and the app freezes. I don't know whether the appointment is booked or not." (RR1) | Rural, low-income, intersectional |
| 4. Reliance on informal support and loss of privacy | Dependence on family/friends/staff; dignity erosion; privacy as luxury | Shared phones/health data exposure; feelings of shame; ad hoc vs structural support | "Mostly family. My children help me. But that means they see my health details. I lose privacy." (OA2) | All groups, especially older adults, rural, disabilities |
| 5. Accessibility, disability, and invisible exclusion | Technical design failures; cognitive overload; neutral design exclusion | Small text/poor contrast; no screen-reader/captions; formal language; multi-step burden | "The text size is small, contrast is poor, and some buttons are not recognized by screen readers." (PD1) | People with disabilities, intersectional |
| 6. Fragile trust and perceived misalignment of equity | State trust vs system distrust; efficiency prioritised over equity | "Hidden line" urban-rural; trust linked to recognition/dignity; neutral = exclusion | "It promotes efficiency, not equity. Those are not the same." (RR3) | All groups, especially rural, intersectional |
| 7. Recommendations for Inclusive Design | Inclusive design features; human/system alternatives; community support | Simplified versions; offline/SMS options; grace periods; training desks/helplines | "A simple version for older adults, with larger text and fewer steps." (OA4) | All groups |
